# Real-Time PCR Detection of Dogwood Anthracnose Fungus in Historical Herbarium Specimens from Asia

**DOI:** 10.1371/journal.pone.0154030

**Published:** 2016-04-20

**Authors:** Stephen Miller, Hayato Masuya, Jian Zhang, Emily Walsh, Ning Zhang

**Affiliations:** 1 Department of Plant Biology & Pathology, 201 Foran Hall, 59 Dudley Road, Rutgers University, New Brunswick, New Jersey, 08901, United States of America; 2 Tohoku Research Center of Forestry & Forest Products Research Institute, 92–25 Nabeyashiki, Shimi-Kuriyagawa, Morioka, Iwate 020–0123, Japan; 3 Chinese Medicine Hospital of Gaomi, Shandong Province, 261500, China; 4 Department of Biochemistry & Microbiology, 76 Lipman Drive, Rutgers University, New Brunswick, New Jersey, 08901, United States of America; Natural Resources Canada, CANADA

## Abstract

*Cornus* species (dogwoods) are popular ornamental trees and important understory plants in natural forests of northern hemisphere. Dogwood anthracnose, one of the major diseases affecting the native North American *Cornus* species, such as *C*. *florida*, is caused by the fungal pathogen *Discula destructiva*. The origin of this fungus is not known, but it is hypothesized that it was imported to North America with its host plants from Asia. In this study, a TaqMan real-time PCR assay was used to detect *D*. *destructiva* in dried herbarium and fresh *Cornus* samples. Several herbarium specimens from Japan and China were detected positive for *D*. *destructiva*, some of which were collected before the first report of the dogwood anthracnose in North America. Our findings further support that *D*. *destructiva* was introduced to North America from Asia where the fungus likely does not cause severe disease.

## Introduction

In North America, several native *Cornus* species, especially *Cornus florida* (flowering dogwood) and *C*. *nuttallii* (Pacific dogwood), have been plagued by the dogwood anthracnose fungus *Discula destructiva* Redlin since the 1970’s [1]. The disease threatens the ecological integrity of forest ecosystems and has caused massive economic losses for the nursery industry [2]. *Cornus florida and C*. *nuttallii* are both widely distributed understory trees in natural forests of the northern hemisphere, which provide food for birds and nutrient recycling through leaf litter [3,4,5]. These two species are members of the big bract morphological group [6] and are closely related to *Cornus* species native to Eastern Asia, such as *C*. *kousa* (Japanese dogwood), which appears to have resistance to the disease. *Cornus florida* and *C*. *kousa* are also valued ornamentals. *Cornus florida* is one of the most popular landscape trees in the United States with $30,901,000 in total sales for 2007 [7].

Dogwood anthracnose was first noted in the west coast of the United States on *C*. *nuttallii* in 1979 [8], and was soon after reported on the east coast [9]. Redlin [1] concluded that isolates from both the east and west coast were morphologically indistinguishable and named the causal agent as a new fungal species, *Discula destructiva*. Several other studies reported that *D*. *destructiva* is distinct from any other North American *Discula* species [10, 11, 12,13]. The disease symptoms include bract necrosis, leaf spot, leaf blight, twig dieback, and trunk canker, which usually start to develop in the spring and early summer. Infected trees in forest settings, where inoculum levels are higher due to shade and moisture, can be killed in as little as one to three years [14]. Since the first reports in the 1970’s, the disease has quickly spread through native North American dogwood populations, from British Columbia to northern California on the west coast and from Vermont to Georgia and Alabama in the east [15]. Within the United States, *D*. *destructiva* has resulted in mortality rates as high as 89% in some forests [16,17]. The disease had not been reported outside North America until 2002 when *D*. *destructiva* was detected on *C*. *florida* in Germany, and also in Italy in 2003 and Switzerland in 2009 [18,19].

The origin of *D*. *destructiva* is unknown but it is hypothesized to be an introduced species, similar to the chestnut blight pathogen [15, 20]. The sudden appearance of the disease near the USA ports, the low genetic variation within the pathogen population [21], and the natural resistance of the native Asian dogwood species suggest that *D*. *destructiva* was introduced, likely from Asia. It is hypothesized that *D*. *destructiva* is an endophyte or latent pathogen and does not typically cause disease on its native host plant when the hosts are not under other biotic or abiotic stresses. However, no previous work has been done on testing the presence of *D*. *destructiva* from dogwoods in Asia.

The identification of *D*. *destructiva* based on the fungal culture morphology and disease symptoms is problematic [22]. This fungus grows slowly on culture media and is often outgrown by the fast growing fungi inhabiting the same host plant tissue. Therefore, a negative detection of *D*. *destructiva* based on culturing is not reliable due to the high likelihood of false negative results. Furthermore, *D*. *destructiva* does not sporulate readily on the conventional media, making morphological identification challenging [14]. Disease symptoms caused by *D*. *destructiva* are similar to those caused by other pathogens such as *Colletotrichum acutatum* [1, 23], making it difficult to accurately detect the pathogen by symptoms alone.

To facilitate the study on the origin and distribution of *D*. *destructiva*, a real-time PCR assay has been developed for fast and accurate detection of this fungus, bypassing the need for culturing [22]. Rapid and reliable detection via real-time PCR is a valuable tool for diagnosing, monitoring, and narrowing down the location of the origin of the species. This assay was applied to test fresh dogwood samples but had not been used to survey dried herbarium specimens [22]. Historical herbarium samples can provide invaluable information about the presence or absence of the fungal species from a long range of time, which allows us to test the hypothesis on the origin of the disease. The objectives of this study were to: (i) test the presence of *Discula destructiva* in the historical herbarium *Cornus* specimens collected from Asia, Europe and North America using real-time PCR assay; and (ii) test the presence of *Discula destructiva* in recently collected fresh dogwood samples from Japan and USA using both culturing and real-time PCR assay.

## Materials and Methods

### Study sites and sampling of fresh *Cornus* species

Samples were collected in late May to early June of 2010 and 2012 from temperate deciduous forests in the United States and Japan. The field studies did not involve endangered or protected species and no specific permissions were required for these locations. Mature leaf samples of wild *C*. *florida* were collected from five trees (13–30 cm diam.; 5–10 m. in height) each year at the Hutchinson Memorial Forest in Somerset, New Jersey, USA (40°30′01″N 74°34′02″W), within the native range of *C*. *florida*. The forest is a nature preserve, one of the few uncut forests in New Jersey [24, 25]. One branch each from the north, west and east-facing sides of the tree was collected from the top of the canopy [26]. Samples were kept at 4°C until DNA isolation. Three leaves from each branch were randomly selected for fungal isolation within three days of sample collection. Following the same sampling strategy, leaf samples of wild *C*. *kousa* were collected from a natural forest in the Ibaraki Prefecture in Japan (36°14′60″N 140°5′20″E), with a similar climate to the United States sampling site. Five trees were sampled and analyzed in each site, each year giving a total of 20 tree samples.

### Herbarium specimens

Seventy herbarium specimens of a variety of *Cornus* species collected from Canada, Mexico, USA, China, Japan, Korea, Nepal, France, and Russia during 1909–2011 were obtained from the New York Botanical Garden and the Harvard Herbarium (Table 1). Required permissions for the specimen sampling were obtained from Dr. Stella Sylva of the New York Botanical Garden and Dr. Michaela Schumll from the Harvard Herbarium.

**Table 1:** Real-time PCR results of *Cornus* herbarium samples with species name, location, and year of collection.

Sample	Plant Species	Location	Year	qPCR mean Ct value (Standard deviation)*
H9	*C*. *alba*	Jilin Province, China	1997	38.0 (0.86)
H38	*C*. *alba*	China	1909	34.0 (0.80)
H42	*C*. *alba*	Montes Kentei, Russia	1929	36.0 (0.65)
H26	*C*. *amomum*	Cayuga Lake, NY	1947	34.7 (0.65)
**H6**	***C*. *brachypoda***	**Nippen, Japan**	**1951**	**31.2 (0.46)**
**H7**	***C*. *brachypoda***	**Hondo, Japan**	**1949**	**31.9 (0.62)**
H8	*C*. *brachypoda*	Nippon, Japan	1951	35.87 (1.02)
H3	*C*. *canadensis*	Prov. Mutsu, Japan	1952	-
H4	*C*. *canadensis*	Pref. Yamanashi, Japan	1959	38.0 (2.07)
H2	*C*. *capitata*	C. Napal, Nepal	1972	-
H10	*C*. *capitata*	Yunnan, China	1946	38.2 (2.25)
H11	*C*. *capitata*	Yunnan, China	2000	38.6 (2.34)
H23	*C*. *chinesis*	Omei-hsien, China	1942	38.1 (0.30)
H24	*C*. *chinesis*	Mt. Omei, China	1938	35.3 (1.70)
**H16**	***C*. *controversa***	**Yubiso, Japan**	**1956**	**31.6 (0.10)**
H18	*C*. *controversa*	Ohshimizu, Japan	1952	-
H19	*C*. *controversa*	Jiangxi, China	1975	41.1 (0.32)
H20	*C*. *controversa*	Nunobiki, Japan	1953	-
H21	*C*. *controversa*	Arima, Japan	1953	-
H22	*C*. *controversa*	Mt. Namari, Japan	1955	-
**H48**	***C*. *florida***	**Carstens, NY**	**2011**	**26.4 (0.07)**
n5	*C*. *florida*	Mississippi, USA	1955	35.5 (0.63)
n6	*C*. *florida*	Louisiana, USA	1971	33.0 (0.34)
n7	*C*. *florida*	NH, USA	1985	32.8 (0.37)
H56	*C*. *japonica*	Huang-shan, China	1979	-
H29	*C*. *kousa*	N. Honshu, Japan	1987	-
H30	*C*. *kousa*	N. Honshu, Japan	1988	38.0 (0.57)
H31	*C*. *kousa*	Yamanashi Pref., Japan	1984	-
H32	*C*. *kousa*	Miyagi Pref., Japan	1984	-
H33	*C*. *kousa*	Fukushima Pref., Japan	1984	36.0 (0.12)
H34	*C*. *kousa*	Yamanashi Pref., Japan	1982	-
H35	*C*. *kousa*	Honshu, Japan	1982	38.5 (1.82)
H36	*C*. *kousa*	Nara Pref., Japan	1978	-
H37	*C*. *kousa*	Shikoku, Japan	1985	36.3 (1.13)
H39	*C*. *kousa*	Mino Pref., Japan	1935	-
H40	*C*. *kousa*	Miyagi Pref., Japan	1988	36.1 (0.75)
H41	*C*. *kousa*	Mt. Ohyama, Japan	1968	36.7 (0.03)
H43	*C*. *kousa*	Montes Kentei, Japan	1965	-
H44	*C*. *kousa*	Tokyo, Japan	1978	35.6 (1.11)
H45	*C*. *kousa*	Tokyo, Japan	1978	-
H46	*C*. *kousa*	Mt. Fuji, Japan	1977	41.3 (2.98)
H47	*C*. *kousa*	M. Hotta, Japan	1965	34.1 (0.47)
H49	*C*. *kousa*	J. Murata, Japan	1976	38.8 (1.67)
H51	*C*. *kousa*	Kukhansan, Korea	1987	35.6 (1.01)
H52	*C*. *kousa*	Yakushima, Japan	1961	36.3 (0.50)
H53	*C*. *kousa*	Hupei-Szechuan, China	1960	36.3 (0.50)
H54	*C*. *kousa*	Isl. Tsushima, Japan	1968	36.7 (0.82)
H55	*C*. *kousa*	Yamanashi Pref., Japan	1982	35.0 (0.74)
H57	*C*. *kousa*	Hupei-Szechuan, China	1960	36.3 (1.45)
H59	*C*. *kousa*	Kyushu, Japan	1983	35.7 (0.13)
H60	*C*. *kousa*	Honshu, Japan	1973	32.2 (0.42)
H61	*C*. *kousa*	Yamanashi Pref., Japan	1976	-
H65	*C*. *kousa*	Shennongjia Forest, China	1980	34.67 (0.87)
H66	*C*. *kousa*	Kiangsi, China	1975	33.5 (0.06)
H27	*C*. *macrophylla*	Metasequoia area, China	1960	-
n1	*C*. *nuttallii*	Oregon, USA	1987	35.4 (1.22)
n2	*C*. *nuttallii*	BC, Canada	1963	-
n3	*C*. *nuttallii*	California, USA	1976	-
n4	*C*. *nuttallii*	Oregon, USA	1975	-
n8	*C*. *nuttallii*	California, USA	1988	33.7 (0.63)
n9	*C*. *nuttallii*	California, USA	1968	33.0 (0.24)
H12	*C*. *paucinervis*	Hupei-Szechuan, China	1974	40.2 (2.50)
H28	*C*. *paucinervis*	Hubei Prov., China	1980	36.2 (1.11)
H25	*C*. *sanguinea*	Nancy, France	1959	34.2 (0.46)
H58	*C*. *stolonifera*	Mt. Potosi, Mexico	1968	-
H64	*C*. *stolonifera*	Nuevo Leon, Mexico	1960	34.35 (0.74)
H62	*C*. *stolonifiera*	Chihuahua, Mexico	1972	36.3 (1.44)
H63	*C*. *stolonifiera*	Chihuahua, Mexico	1977	33.5 (0.38)
**H13**	***C*. *walteri***	**Sichuan Prov., China**	**2007**	**31.2 (0.28)**
**H14**	***C*. *walteri***	**Hupeh:Shenlungkai, China**	**1976**	**31.1 (0.16)**

*Highlighted in bold are the samples with positive detection of *Discula destructiva*, with Ct value < 32.0, and confirmed with DNA sequencing. -: no amplification signals.

### Plant DNA extraction, real-time PCR and sequencing

For each sample, 50 mg leaf tissue was ground in liquid nitrogen. Genomic DNA was then isolated using Qiagen DNeasy Plant Mini kit (Qiagen, Germany) following the manufactures protocol (Tables 1 and 2). All real-time PCR reactions were performed on the StepOnePlus real-time PCR system (Applied Biosystems, CA, USA) following the procedures described in our previously published paper [22]. Primers used for the detection of *D*. *destructiva* were DdITS_F1 and DdITS_R1, along with the probe DdITS_Probe1 [22]. The following conditions were used to carry out the real-time PCR reaction: 3 min of 95°C, followed by 45 cycles of 15 s at 95°C, and 40 s at 60°C. Each reaction consisted of 10 μl iTaq Supermix with ROX (Bio-Rad, CA, USA), 250 nM probe, 500 nM of each primer, and 4 μl template DNA for a total volume of 20 μl. A standard curve was also constructed using genomic DNA from *D*. *destructiva* isolate MD235, which is a type culture of this species [1]. A Ct value of less than 32 was counted as positive detection of *D*. *destructiva*. Each sample was tested in triplicate. The real-time PCR products were further subjected to purification using QIAquick PCR purification kit (Qiagen, CA), following the manufacturers protocol. The purified amplicons were sequenced by GeneWiz, Inc. (South Plainfield, NJ, USA) with primers DdITS_F1 and DdITS_R1 to confirm the identity of the amplified sequences.

**Table 2:** Real-time PCR detection results of fresh *Cornus* leaf samples collected from New Jersey, USA and Ibaraki Prefecture, Japan in 2010 and 2012.

Sample	Plant Species	Location	Year	qPCR mean Ct value (Standard deviation)*
US10-1	*C*. *florida*	USA	2010	-
US10-2	*C*. *florida*	USA	2010	-
**US10-3**	***C*. *florida***	**USA**	**2010**	30.6 (1.90)**
US10-4	*C*. *florida*	USA	2010	34.9 (0.60)
**US10-5**	***C*. *florida***	**USA**	**2010**	17.01 (0.43)**
US12-1	*C*. *florida*	USA	2012	-
US12-2	*C*. *florida*	USA	2012	-
US12-3	*C*. *florida*	USA	2012	-
US12-4	*C*. *florida*	USA	2012	-
US12-5	*C*. *florida*	USA	2012	-
**JAP10-1**	***C*. *kousa***	**Japan**	**2010**	**23.9 (0.42)**
JAP10-2	*C*. *kousa*	Japan	2010	-
JAP10-3	*C*. *kousa*	Japan	2010	-
JAP10-4	*C*. *kousa*	Japan	2010	-
JAP10-5	*C*. *kousa*	Japan	2010	38.3 (0.46)
JAP12-1	*C*. *kousa*	Japan	2012	38.2 (1.43)
JAP12-2	*C*. *kousa*	Japan	2012	34.8 (0.76)
JAP12-3	*C*. *kousa*	Japan	2012	35.0 (0.59)
JAP12-4	*C*. *kousa*	Japan	2012	34.1 (0.91)
JAP12-5	*C*. *kousa*	Japan	2012	34.8 (0.76)

*Highlighted in bold are the samples with positive detection of *Discula destructiva*, with Ct value < 32.0, and confirmed with DNA sequencing. -: no amplification signals.

** Cultures of *Discula destructiva* were also obtained from these samples.

### Fungal isolation and morphological identification

For the fresh *Cornus* leaf samples collected from USA and Japan, three leaves from each branch sampled from each tree were cut along various sections of the leaf (lamina tip, lamina margin to midrib, and lamina base) into multiple 0.5 cm segments that were surface-sterilized through sequential immersion in 0.5% sodium hypochlorite, 70% (v/v) ethanol, and rinsed three times in sterilized distilled water [27, 28]. Leaf segments were air dried and placed on Petri dishes containing 2% acidified MEA (AMEA). One liter AMEA contained 20 g malt extract (BD Biosciences, Sparks, MD), 20 g agar (BD Biosciences, Sparks, MD) and 1 ml of 85% lactic acid (Sigma-Aldrich, St. Louis, MO, USA). Five leaf segments from each sample were placed on a control AMEA plate for 30 seconds and then removed [27, 28, 29], which were used to monitor for epiphytic fungal growth. Petri dishes were incubated for six months under room temperature (22–24°C).

Emerging colonies were sub-cultured to obtain pure fungal isolates. After a week, subcultures growing in AMEA were grouped into morphotaxa [28, 30, 31] based on spore morphology (if present), as well as colony characteristics such as shape, color, texture, aerial hyphae, and margin. If more than three isolates were present in a morphotaxon, three representative isolates were selected for sequencing. If there were fewer than three isolates in a morphotaxon, all isolates were sequenced.

### Fungal DNA extraction, PCR, sequencing and identification

Fungal isolates were grown on AMEA at room temperature for four days to two weeks depending on growth rate. Genomic DNA was extracted from mycelium with Qiagen DNeasy Plant Mini kit (Qiagen, Germany) following the manufacturer’s protocol. The internal transcribed spacer (ITS) of the rRNA genes was amplified with ITS1 and ITS4 primers [32]. ITS1F was used with ITS4 if no PCR product was found with ITS1 and ITS4. PCR reaction mixture (25 μl) consisted of 5 μl of 5X GoTaq Flexi Buffer (Promega, WI, USA), 1.5 μl of 25 mM MgCl_2_, 2 μl of 10 mM dNTPs mix, 1 μl of 10 mM forward primer and 1 μl of 10 mM reverse primer, 0.125 μl (5U/μl) of GoTaq DNA polymerase (Promega, WI, USA), and a maximum of 25 ng/μl of genomic DNA. The PCR cycling conditions were as follows: 94°C for 5 minutes, followed by 32 cycles of denaturation at 95°C for 1 minute, annealing at 55˚C for 1 minute, and primer extension at 72°C for 1.5 minutes, followed by a final extension at 72°C for 5 minutes. PCR products were verified using gel electrophoresis and purified using ExoSAP-IT (USB Corporation, Cleveland, OH, USA) following the manufacturer’s instructions. Purified PCR products were sequenced by GeneWiz, Inc. (South Plainfield, NJ, USA) using primers ITS1, ITS4 and ITS1F [32].

Fungal isolates were identified based on morphology and Mega BLASTn for each of the ITS sequences against GenBank. The ITS sequences (ca. 500 bp) were compared against a curated GenBank database using their Mega BLAST program on a local server. Top sequences that matched >97% similarity were considered belonging to the same operational taxonomic unit (OTU). DNA sequences obtained in this study are deposited in GenBank under accession numbers KJ921855-KJ921969.

## Results

### Real-time PCR results

Six herbarium samples (H6, H7, H13, H14, H16 and H48) (Table 1) had Ct values below 32, the cutoff Ct value, which was decided based on the positive and negative control real-time PCR readings, and the sequencing results of the real-time PCR amplicons. Samples H6, H7, H13, H14, and H16 were from various *Cornus* species located in Japan and China collected during 1949–2007. H48 was collected in USA in 2011. The real-time PCR assay also detected *D*. *destructiva* from three recently collected fresh samples from USA and Japan (Table 2). All samples with positive real-time PCR detection results were verified to have a 191 bp amplicon, the sequence of which matched to the ITS sequence of *D*. *destructiva* (100% identity).

### *Cornus* endophyte analysis

A total of 371 fungal culture isolates were obtained from 1825 leaf segments from the 20 fresh *Cornus* samples collected from USA and Japan in 2010 and 2012, and 121 representative isolates were sequenced. A total of 48 OTUs were identified that belong to 26 genera (S1 Table). There was no fungal growth present on the control plates after surface sterilization of the leaf samples. *Discula destructiva* was isolated in culture from the USA samples but not from the samples from Japan.

## Discussion

The origin of the dogwood anthracnose pathogen had been a mystery [15]. The current hypothesis is that the fungus was introduced from Asia to North America in the 1970’s [15, 20]. It has later spread into Europe due to trade [22]. However, there had been no report on the presence of this fungal species in Asia. This study is the first time report of positive detection of *D*. *destructiva* from dogwood samples in Asia. The fact that some of the *D*. *destructiva*-positive herbarium samples were collected in Asia before the first disease outbreak in North America provides evidence for the introduction hypothesis.

A major challenge in searching for the dogwood anthracnose fungus and other slow-growing microscopic species is a lack of accurate and sensitive detection methods. The real-time PCR assay allowed for sensitive and reliable testing for both fresh [22] and herbarium samples. In this study, we successfully detected the dogwood anthracnose fungus from dried specimens collected up to 66 years ago. The results here present an early attempt to utilize this molecular method to detect fungal endophytes or pathogens from historical herbarium samples.

A live culture of *D*. *destructiva* has not been obtained from Asia, likely due to its slow growth rate in culture and the small number of fresh samples included in this study. The positive molecular detection from Asia indicates that further sampling efforts in Japan and other areas of Asia likely will yield *D*. *destructiva* cultures, which will provide long-awaited materials for future studies, in order to better understand the origin, dispersal and evolution of this *Cornus*-associated fungus.

## Supporting Information

S1 Table List of identified fungal OTUs and their abundance by year and location. (DOCX)
