# Supplementary material for: Real-Time PCR Detection of Dogwood Anthracnose Fungus in Historical Herbarium Specimens from Asia
Source: PLoS One. 2016 Apr 20;11(4):e0154030. doi: 10.1371/journal.pone.0154030 (PMC4838270; doi:10.1371/journal.pone.0154030)
Supplement: S1 Table — (DOCX) [file pone.0154030.s001.docx]

| Table S1. List of identified fungal OTUs and their abundance by year and location | | | | |
| --- | --- | --- | --- | --- |
|  | 2010 USA | 2010 Japan | 2012 USA | 2012 Japan |
| Alternaria alternata | 4 | 3 | 4 | 1 |
| Alternaria tenuissima | 0 | 0 | 0 | 1 |
| Phoma peniodella | 0 | 0 | 2 | 0 |
| Phoma glomerata | 1 | 3 | 3 | 4 |
| Lophiostoma sp. | 1 | 0 | 0 | 0 |
| Dothideomycetes sp. | 0 | 0 | 3 | 0 |
| Botryosphaeria_dothidea | 0 | 2 | 0 | 4 |
| Botryosphaeria_sp. 1 | 0 | 1 | 0 | 0 |
| Cladosporium sp. | 0 | 1 | 0 | 1 |
| Aureobasidium pullulans | 2 | 0 | 1 | 0 |
| Aureobasidium sp. 2 | 3 | 0 | 1 | 0 |
| Elsinoe fawcettii | 10 | 0 | 8 | 0 |
| Aspergillus sp. | 2 | 1 | 1 | 1 |
| Penicillium cecidicola | 0 | 1 | 0 | 2 |
| Penicillium chrysogenum | 2 | 0 | 1 | 0 |
| Penicillium janthinellum | 2 | 0 | 2 | 0 |
| Penicillium spinulosum | 0 | 2 | 0 | 2 |
| Colletotrichum acutatum | 2 | 4 | 26 | 28 |
| Colletotrichum sp. 1 | 0 | 0 | 0 | 2 |
| Verticillium dahlaie | 3 | 0 | 0 | 0 |
| Trichoderma lixii | 6 | 9 | 15 | 10 |
| Neonectria discophora | 0 | 2 | 0 | 0 |
| Neonectria sp 1 | 0 | 1 | 0 | 0 |
| Neonectria sp 2 | 1 | 0 | 0 | 0 |
| Fusarium sp.1 | 0 | 0 | 0 | 1 |
| fusarium vilior | 4 | 3 | 9 | 7 |
| Discula Destructiva | 1 | 0 | 1 | 0 |
| Pleuroceras tenellum | 9 | 0 | 0 | 0 |
| Tubakia sp. | 0 | 0 | 3 | 6 |
| Phomopsis endophytica | 1 | 0 | 0 | 0 |
| Phomopsis lagerstroemiae | 0 | 1 | 0 | 2 |
| Phomopsis amygdali | 3 | 1 | 2 | 1 |
| Phomopsis vaccinii | 0 | 2 | 0 | 3 |
| Phomopsis nobilis | 1 | 1 | 1 | 2 |
| Phomopsis sp. 1 | 1 | 1 | 1 | 0 |
| Phompsis sp. 2 | 2 | 0 | 3 | 0 |
| Xylariales sp. | 0 | 0 | 3 | 0 |
| Xylaria submonticulosum | 2 | 1 | 3 | 1 |
| Xylaria sp. 1 | 1 | 0 | 0 | 0 |
| Xylaria sp. 2 | 2 | 2 | 3 | 3 |
| Cyrptosporiopsis sp. | 0 | 0 | 2 | 0 |
| Neofabrea sp. | 2 | 0 | 0 | 0 |
| Pestalotiopsis mangiferae | 1 | 0 | 2 | 0 |
| Pestalotiopsis microspora | 25 | 16 | 18 | 12 |
| Pestalotiopsis monochaeta | 0 | 1 | 0 | 0 |
| Pestalotiopsis sp. 1 | 0 | 2 | 0 | 1 |
| Pestalotiopsis sp. 2 | 0 | 1 | 0 | 1 |
| Pestalotiopsis sp. 3 | 0 | 1 | 0 | 1 |
